# Analytical evaluation of the new Seal Autoanalyzer 3 High Resolution for urinary iodine determination

**DOI:** 10.11613/BM.2019.020711

**Published:** 2019-06-15

**Authors:** Valentina Vidranski, Maja Franceschi, Dražena Krilić, Tomislav Jukić, Ivan Mihaljević, Zvonko Kusić

**Affiliations:** 1 Department for Oncology and Nuclear Medicine, University Hospital Center Sestre milosrdnice, Zagreb, Croatia; 2 Faculty of medicine Osijek, Josip Juraj Strossmayer University of Osijek, Osijek, Croatia; 3 Department for Nuclear Medicine and Radiation Protection, University Hospital Center Osijek, Osijek, Croatia; 4 Croatian Academy of Sciences and Arts, Zagreb, Croatia

**Keywords:** colorimetry, iodine, method evaluation, urine

## Abstract

**Introduction:**

The aim of the study was to evaluate the analytical performance of the new colorimetric, automatic analyser, Seal AutoAnalyzer 3 High Resolution (Seal AA3 HR) (Seal Analytical, Wisconsin, USA) for urinary iodine measurement.

**Materials and methods:**

This study included testing of several analytical features of the method involving: imprecision (within-run %CVr, between-run %CVb and total laboratory precision %CVl), measurement uncertainty, carryover, linearity and method comparison, with 70 urine samples including the measuring range (20 - 700 µg/L).

**Results:**

Within-run, %CVb and %CVl of two control levels were 2.03% and 3.04%, 0.51% and 2.61%, and 2.09% and 4.01%, respectively. Carryover effect was less than 1%. The linearity was good in the range of urinary iodine values between 60 and 500 µg/L (R^2^ = 0.99). Good agreement of urinary iodine values was found between manual technique and Seal AA3 HR, using Passing-Bablok regression (y = 7.84 (- 3.00 to 15.29) + 0.95 (0.90 to 1.00) x) and Blant-Altman test. Cusum test for linearity indicates that there is no significant deviation from linearity (P > 0.1).

**Conclusions:**

The obtained results proved excellent precision, reproducibility and linearity, comparable to the already used, manual method. The New Seal AA3 HR automatic analyser is acceptable for urinary iodine measurement with very good analytical characteristics and can be used for urinary iodine epidemiological studies of the Croatian population.

## Introduction

Iodine is an essential component of thyroid hormone synthesis and can be normally obtained only through food consumption. Thyroid hormones, among all, have a key function in the regulation of the growth and development of the foetus and child as well as in metabolism, temperature regulation and blood circulation (*1*–*3*).

Iodine deficiency as well as iodine excess, is a global health problem. It causes complications with the woman’s reproductive cycle, complications with pregnancy, irreversible congenital anomalies of new-borns and thyroid dysfunction in adults. To ensure that everyone has a sufficient intake of iodine, The World Health Organization (WHO) and United Nations International Children’s Emergency Fund (UNICEF) recommend universal salt iodization as a global strategy (*4*–*8*).

Around 90% of daily iodine intake is excreted through urine, a negligible part through sweat, the stool and breast milk in lactating women, so urinary iodine is the perfect biochemical parameter that reflects the iodine status population. Urinary iodine concentration 100-300 µg/L meets adequate iodine assessment, below 100 µg/L is defined as iodine deficiency (< 20, 20-49 and 50-99, severe, moderate and mild) and above 300 µg/L, as excessive. Pregnant and lactating women as well as children aged 2 and less have different iodine requirements (*9*, *10*).

Urinary iodine determination methods are numerous, all quantifying small urine amounts. There are two main methodologies: the spectrophotometric one, based on the Sandell-Kolthoff (SK) reaction and Inductively Coupled Plasma-Mass Spectrometry method (ICP-MS). Pre-analytical, analytical and post-analytical goals, according to quality, can be achieved by the both methods.

The Sandell-Kolthoff reaction, described in 1937, is based on the catalysm of iodide and reduction of ceric ammonium sulphate to the cerous form, in the presence of arsenious acid and it’s the most common way for urinary iodine measure. The choice of the method depends on the country’s needs and financial capabilities since there is no standardized method for urinary iodine determination although ICP-MS is the best sophisticated method (*3*, *11*, *12*).

Seal Analytical has introduced an alternative colorimetric analyser for in vitro diagnostics of urinary iodide, The Seal AutoAnalyzer 3 High Resolution (Seal AA3 HR) (Seal Analytical, Wisconsin, USA). It is the new generation of the original Technicon AutoAnalyzer (Technicon Corporation Bayer, NY, USA).

The method is commercially available on The Seal AA3 HR analyser but, as we know, we are the only users and it’s made for our purposes so it’s a unique combination of the specific method for urinary iodide measurement of the mentioned analyser. The aim of the study was to evaluate the analytical performance of the new colorimetric, analyser, the Seal AA3 HR for urinary iodine measurement, the testing of several analytical features of the method involving: imprecision (within-run %CV_r_, between-run %CV_b_ and total laboratory precision %CV_l_), measurement uncertainty, carryover, linearity and method comparison.

## Materials and methods

### Study design

This analytical verification was conducted at the Department of Oncology and Nuclear medicine, University Hospital Center Sestre milosrdnice, Zagreb, Croatia, during January, August and September 2018.

This study was part of an epidemiological study in Croatia from The Croatian Science Foundation, grant IP-2014-09-6499 that had approval from The Ethical Committee of The School of Medicine, at The University of Zagreb and the University Hospital Center Sestre milosrdnice.

### Materials/Subjects

Urine samples were collected on a school day, from 6 - 12 year olds, apparently healthy primary school, children comprised of both males and females from Zagreb, Croatia during 2015 and 2016. Their anthropometric measurements and thyroid ultrasound were taken.

The first urine samples were collected into sterile cups for the urine collection (Greiner Bio-One, Kremsmünster, Austria) and aliquoted into tubes. Since the samples were not analysed immediately, the three urine aliquots (2 mL) were stored at - 20 °C until their analysis.

One aliquot was defrosted and processed by a manual method for research purposes and the result was included in the statistical analysis of the National study. Other samples are residual quantities and stayed frozen until analysis. Aliquot was defrosted by standing at room temperature until liquid state. Urine samples were carefully chosen according to the obtained results.

During collection of urine samples, parents signed an Inform consent.

Reagents were prepared according to manufacturer instructions. Following reagents were used on the analyzer: ammonium cerium (IV) sulphate dehydrate, arsenic trioxide _,_ potassium iodide, Brij-35 solution, 22-30% solution, phosphoric acid, conc., potassium persulfate, sodium chloride, sodium hydroxide and sulphuric acid, conc. 98%, H_2_SO_4_, all Merck (Merck, Darmstadt, Germany). Ultra-high purity water was used for the preparation of reagents, the purification system NIRO VV 40 LAB (Nirosta d.o.o. Water Technologies, Osijek, Croatia).

Potassium iodide stock standard A (100 mg/dL), stock standard B (1 mg/dL) and working standard (50 µg/dL) (Merck, Darmstadt, Germany) for standard preparation (Calibrator 1: 31.25 µg/L, Calibrator 2: 62.5 µg/L, Calibrator 3:125 µg/L, Calibrator 4: 250 µg/L and Calibrator 5: 500 µg/L).

### Methods

The Seal AA3 HR is designed specifically for colorimetric determination of dissolved micronutrients in environmental samples. It runs 40 samples *per* hour.

Until now, the laboratory in our Department used the SK reaction in one variation of the manual method for epidemiology studies of iodine deficiency in The Republic of Croatia. The manual method uses a heating block and spectrophotometric determination of iodine and only 16 to 32 samples of urine could be done daily. The manual method has an open source of toxic and carcinogenic arsenious acid, and other lung dangerous chemicals and it is demanding with regards to time. A stopwatch was used to keep a constant interval between the additions of reagents (30 seconds was a convenient interval). The reduction of iodine was read spectrophotometrically at 420 nm against the reagent blank at the same interval requirements (*13*).

A basic model of The Seal AA3 HR consists of an auto-sampler, a peristaltic pump, a chemistry manifold, a detector and data acquisition software. The peristaltic pump represses samples and reagents continuously through tubes and a chemical manifold. Air bubbles are introduced at regular intervals forming unique reaction segments, which are mixed using small amounts of detergent in glass coils.

The coils are made of glass because glass is an ideal material, due to inertia, and easy visual checking. It has 13 pump tubes, 3 air tubes and one sampler wash tube. The sample stream is mixed with sulphuric acid and passes through a quartz coil in the UV digestor where protein-bound iodine is released as iodide. The digested sample is mixed with arsenious acid and ceric ammonium sulphate solution. The reaction mixture passes through a heating bath (55 °C) to ensure complete reaction.

The yellow Ce(IV) is reduced to colourless Ce(III) and the reduction in colour intensity is measured at 420 nm.

As^3+^ + I_2_ → As^5+^ + 2I^-^

2Ce^4+^ + 2I^-^ → 2Ce^3+^ + I_2_

(yellow) (colourless)

The Seal provides a very detailed, user-friendly brochure to provide information about the Seal AA3 HR, and has very good customer support (*14*).

For imprecision testing quality control samples ClinCheck were used, for trace elements (Recipe, Munich, Germany). Controls are lyophilized, based on human urine and we have had two concentrations of control of the same lot (*15*).

For external quality assurance of urinary iodine concentration, our laboratory is included in Ensuring the Quality of Iodine Procedures (EQUIP) program by The Centers for Disease Control and Prevention (CDC), Houston, USA. Countries with urinary iodine deficiency national surveys participate, three times a year, free of charge (*16*).

### Precision

Controls were tested in triplicate for five days in accordance with CLSI EP15-A2 protocol, N = 30 (*17*). The coefficient of variation, CV% is calculated to a standard deviation using the overall, grand mean (arithmetic mean of single day means).

Repeatability as %CV_r_, reproducibility %CV_b_ and within-laboratory precision %CV_l_, were performed using two commercial control materials: low (mean = 122.17 µg/L) and high (mean = 487.6 µg/L) control concentration.

Within-laboratory precision represents the relative standard measurement uncertainty (u_rel_). Expanded measurement uncertainty (U_rel_) is calculated as U_rel_ = u_rel_ x 2 and represents the 95% confidence interval of measured results.

The Royal College of Pathologists of Australasia (RCPA) and The Australasian Association of Clinical Biochemists (AACB) provide guidelines for trace elements acceptable performance that can be used as Analytical Quality Requirements, reviewed in April 2012. According to them, to external quality assesment (EQA), allowable limit of performance for urinary iodine is ± 0.08 till 0.80 µmol/L, then 10% > 0.8 µmol/L (*18*). It was first the allowable limit that we considered, and it is the same as the second one that is according to The Seal Analytical which declines 10% of the allowable precision presented as CV%.

### Carryover

We tested the carryover for three days, using two specimens from donors, one low (below 50 µg/L) and the other with high urinary iodine concentration (above 300 µg/L) according to WHO guidelines for iodine assessment (*10*).

The specimens were divided into 3 tubes, two low and one high. Low urinary concentration specimens were analysed before and after analysing the high urinary iodine concentration specimen. The measurements were carried out once a day, during the 3-day period in triplicate and average values were used for calculation (*19*, *20*).

The carryover bias was calculated as follows: [(mean of low value_(after high sample)_ – mean of low value_(before high sample)_) / mean of low value_(before high sample)_] x 100.

### Linearity

A quantitative analytical method is linear when there is a mathematically verified straight-line, between the observed values and the true concentrations or activities of the analyte. The manufacturer has declared that the linearity range of urinary iodine assay 0 - 500 µg/L and we consider R > 0.99 as linear.

The linearity study was assessed to verify the linear measuring interval of a measurement for a measuring system according to the CLSI guideline EP06-A “Evaluation of the Linearity of Quantitative Measurement Procedures” (*21*).

Three dilutions were prepared by mixing the high and low concentration sample as ratios: 1:4, 1:1 and 4:1. The dilution and each sample concentration were analysed in duplicate. The observed values were plotted against the expected values.

### Methods comparison

The methods comparison was conducted according to the CLSI guideline EP09-A3 using 70 human samples in a range from 30 to 700 µg/L (*11*). The goal was to include samples with specific concentrations to cover the measurement range (*22*). The samples were first determined by a manual technique.

For method comparison we used Passing–Bablok regression to determine systematic error and Bland-Altman plot to assess bias across the measurement range. Passing-Bablok regression is displayed as (y = a (95% CI) + b (95% CI) x), proportional (regression line’s slope (b)) or constant (regression line’s intercept (a)) (*23*).

### Statistical analysis

The statistical analysis was performed using MedCalc statistical software 10.4 (MedCalc software, Ostend, Belgium) for method comparison and Microsoft Office Excel 2010 (Microsoft, Washington, USA) for method comparison, linearity and carryover. Neither one result of the measuring imprecision were not eliminated according to Grubb’s formula. For method comparison, we used Passing–Bablok regression to determine systematic error and Bland-Altman plot to assess bias across the measurement range.

## Results

### Precision study

Table 1 shows that the grand mean of control samples for control 1 was 122.17 µg/L and for control 2 was 418.67 µg/L. Within-run precision (control 1 = 2.03% and control 2 = 3.04%) and between-run precision (control 1 = 0.51% and control 2 = 2.61%) was obtained. The standard deviation in µg/L within laboratory (control 1 = 2.56 and control 2 = 19.54), within laboratory precision (control 1 = 2.09% and control = 24.01%), precision according to Seal (%CV = 10%) and precision according to EQA (CV = 10%) as well as measurement uncertainty (control 1 = 4.18% and control = 28.02%) was obtained.

**Table 1:** Verification results and desirable specifications for imprecision

	**Grand mean, μg/L**	**SD_r_, μg/L** **(%CV_r_)**	**SD_b_, μg/L** **(%CV_b_)**	**SD_l_, μg/L** **(%CV_l_)**	**Seal** **%CV**	**EQA %CV**	**%U_rel_**
**control 1**	122.17	2.48 (2.03%)	0.62 (0.51%)	2.56 (2.09%)	10	10	4.18
**control 2**	487.60	14.82 (3.04%)	12.75 (2.61%)	19.54 (4.01%)	10	10	8.02
Grand mean - arithmetic mean of all measurement (each control). SD_r_ - standard deviation intra-assay. %CV_r_ - within run precision-repeatability. SD_b_ - standard deviation between run. %CV_b_ - between run precision (day to day) – reproducibility. SD_l_ - standard deviation within laboratory. %C_l_ - within laboratory precision. Seal %CV - precision given by the manufacturer. EQA - External Quality Assessment according to RCPA Quality Assurance Programs (allowable limits of performance). %U_rel_ - measurement uncertainty.							

### Carryover study

Table 2 shows carryover testing, between high and low urinary iodine concentrations. According to the formula, the calculated carryover was 0.3%.

**Table 2:** Carryover testing results on Seal AA3 HR analyser

**Urine sample**	**1st day** **UI, µg/L**	**2nd day** **UI, µg/L**	**3rd day** **UI, µg/L**
**1st cup low, 1st time**	38.1	36.5	37.8
**1st cup low, 2nd time**	39.2	38.2	38.1
**1st cup low, 3rd time**	35.6	37.4	37.6
**1st cup high, 1st time**	503.6	490.3	492.1
**1st cup high, 2nd time**	485.7	492.4	489.5
**1st cup high, 3rd time**	490.6	495.4	492.4
**2nd cup low, 1st time**	38.4	37.6	38.4
**2nd cup low, 2nd time**	37.6	36.8	36.1
**2nd cup low, 3rd time**	38.2	37.5	38.9
UI - Urinary iodine. low - sample with low urinary iodine concentrations. high - sample with high urinary iodine concentrations.			

### Linearity

**Figure 1:**
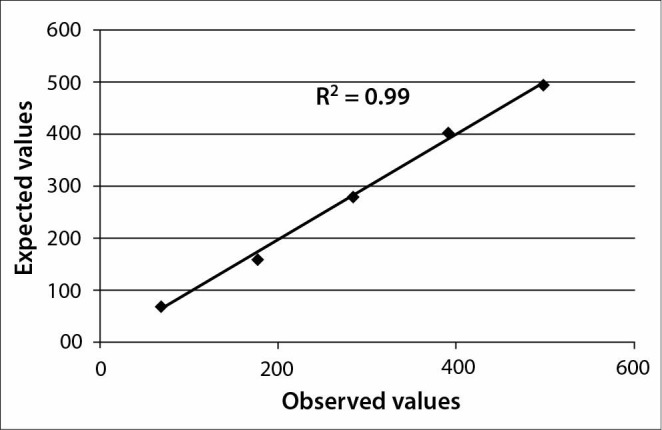
Plot of observed and expected urinary iodine values (μg/L) in linearity study. R^2^ - coefficient of determination.

Table 3 and Figure 1 show the linearity data. The linearity was demonstrated throughout the measuring range, from 60 to 500 µg/L, with R^2^ of 0.99.

**Table 3:** Expected and observed linearity data on Seal AA3 HR

**Sample**	**Sample** **ratio**	**1st measure,** **µg/L**	**2nd measure,** **µg/L**	**Expected value, µg/L**	**Observed value, µg/L**	**Bias, %**
L	1	503.6	485.7	499.0	494.7	- 0.87
L:N	1:4	407.9	400.5	391.3	404.2	3.31
L:N	1:1	279.5	282.4	283.5	281.0	- 0.90
L:N	4:1	158.0	161.2	175.8	159.6	- 9.17
H	1	67.5	70.0	68.0	68.8	1.13
L – sample with low urinary iodine concentration in µg/L. H – sample with high urinary iodine concentration in µg/L						

### Method comparison study

**Figure 2:**
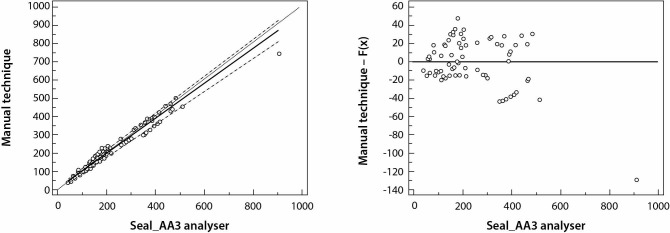
Passing-Bablok regression plots comparing urinary iodine concentrations (μg/L) of manual technique and analyser Seal AA3 HR. Solid line - regression line. Dashed lines - 95% confidence interval of the regression line. Dotted line - identity line (Y=X).

**Figure 3:**
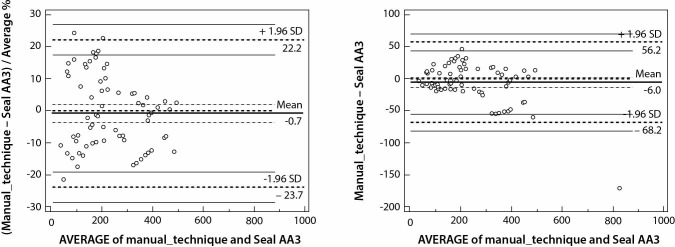
Bland-Altman plot with representation of the limits of agreement. Solid line - mean difference. Dashed lines (SD) - standard deviation.

The Passing-Bablok regression data shown as y = 7.84 (- 3.00 to 15.29) + 0.95 (0.90 to 1.00) x in Figure 2 where x-plot shows urinary iodine concentrations (µg/L) determined by the manual technique and y-plot urinary iodine concentration on analyser Seal AA3 HR. The Bland-Altman plot for method comparison, where x-plot presents urinary iodine (μg/L) by manual technique and y-plot differences between measurements of two methods, is shown in Figure 3.

## Discussion

When introducing a new analyser to the laboratory system, the evaluation of analytical performance (verification) is an important requirement due to standards in laboratory medicine. There are various methods for urinary iodine determination, but the verification data of those is insufficient and according to current literature does not involve CLSI guidelines (*13*, *24*–*29*). Gnat and colleges verified fast colorimetric manual method compering it to older version of our automatic analyser, Technicon AutoAnalyzer II (AA2) but logaritm of determinated data was different (*24*). May and authors evaluated automated method on AA2 system comparing with manual alkaline ashing technique and problems of interfering substances in urinary iodine determination (*25*). Paired-ion-RP HPLC method and AA2 but with ammonium persulfate as oxidant, were described by Bier et al (*26*). Neither one of the above weren’t followed by CLSI guidelines for analytical verification and didn’t involve Seal AA3 HR.

By conducting an analytical evaluation of the new Seal AA3 HR, our aim was to examine whether a new analyser could be implemented in routine laboratory work instead of the used manual technique. The precision of the test results, shown as repeatability, reproducibility and total laboratory precision showed that all obtained values meet the eligibility criteria declared by the manufacturer. The manufacturer also listed the chemicals and the desired analytical reagent-grade, but did not specify the reagent manufacturer itself. Our selection of the chemicals manufacturer and their dissolution proved to be good. It should be emphasized, that the precision, depends solely on random error distribution and does not provide information about the accuracy and true value. The increase in the standard deviation value and the coefficient of variation indicates an increase in measurement imprecision.

There was “carryover” effect below 1%, which is acceptable and is not clinically relevant. The test linearity of the method was very good, since the coefficient of determination, R^2^ was equal to 0.99.

Analyses of 70 donor samples were performed to test the accuracy study comparison of the new analyzer and the used manual technique. The manual technique was compared with the Seal AA3 HR on the basis of the slope and intercept didn’t show constant or proportional bias between the calculated and measured concentrations. Regression showed good concordance. The Bland-Altman plot showed the mean difference, bias - 6.1 and the agreement limits from - 68.2 to 56.2, and plot in percentage difference is about ± 20%, which is acceptable according to EQUIP. The Cusum test for linearity indicates that there is no significant deviation from linearity.

With this analytical evaluation, we proved that the new automatic analyser, Seal AA3 HR is acceptable for laboratory work with good analytical characteristics. The measured imprecision, carryover and linearity were acceptable. The Seal AA3 HR is comparable to the already used, manual method.

The analyser is user friendly, simple, time-consuming and most importantly health friendly due to the closed bottle of highly toxic arsenic acid.

## References

[1] World Health Organization (WHO). Iodine status worldwide: WHO global database on iodine deficiency. Geneva: WHO; 2004. Available at: http://whqlibdoc.who.int/publications/2004/9241592001.pdf. Accessed February 14th 2019.

[2] Eastman CJ, Zimmermann MB. The Iodine Deficiency Disorders. [Updated 2018 Feb 6]. In: Feingold KR, Anawalt B, Boyce A, et al., eds. Endotext [Internet]. Available at: https://www.ncbi.nlm.nih.gov/books/NBK285556/. Accessed February 12th 2019.

[3] Laurberg P, Pedersen IB, Carlé AS, editors. The U-Shaped Curve of Iodine Intake and Thyroid Disorders. In: Comprehensive Handbook of Iodine. Preedy VR, Burrow GN, Watson R, eds. Available at: https://www.sciencedirect.com/book/9780123741356/comprehensivehandbook-of-iodine. Accessed August 15th 2018. https://doi.org/10.1016/b978-0-12-374135-6.00047-9

[4] Wainwright P Cook P The assessment of iodine status – populations, individuals and limitations. Ann Clin Biochem. 2019;56:7–14. 10.1177/0004563218774816 29703103

[5] Luo Y Kawashima A Ishido Y Yoshihara A Oda K Hiroi N Iodine excess as an environmental risk factor for autoimmune thyroid disease. Int J Mol Sci. 2014;15:12895–912. 10.3390/ijms150712895 25050783 PMC4139880

[6] Leung AM Braverman LE Consequences of excess iodine. Nat Rev Endocrinol. 2014;10:136–42. 10.1038/nrendo.2013.251 24342882 PMC3976240

[7] Sun X Shan Z Teng W Effects of increased iodine intake on thyroid disorders. Endocrinol Metab (Seoul). 2014;29:240–7. 10.3803/EnM.2014.29.3.240 25309781 PMC4192807

[8] Bürgi H Iodine excess. Best Pract Res Clin Endocrinol Metab. 2010;24:107–15. 10.1016/j.beem.2009.08.010 20172475

[9] Leung AM Braverman LE He X Heeren T Pearce EN Breastmilk iodine concentrations following acute dietary iodine intake. Thyroid. 2012;22:1176–80. 10.1089/thy.2012.0294 23050787 PMC3487113

[10] Hays MT Colonic excretion of iodide in normal human subjects. Thyroid. 1993;3:31–5. 10.1089/thy.1993.3.31 8499762

[11] World Health Organization (WHO). Vitamin and Mineral Nutrition Information System. Urinary iodine concentrations for determining iodine status in populations 2013. Available at: http://apps.who.int/iris/bitstream/handle/10665/85972/WHO_NMH_NHD_EPG_13.1_eng.pdf;jsessionid=87ADA00C527F6ADED8DE25115675C139?sequence=1%0Ahttp://apps.who.int/iris/bitstream/10665/85972/1/WHO_NMH_NHD_EPG_13.1_eng.pdf. Accessed September 6th 2018.

[12] Sandell EB Kolthoff IM Micro determination of iodine by a catalytic method. Mikrochim Acta. 1937;1:9–25. 10.1007/BF01476194

[13] Urinary iodine methods. Available at: http://www.iccidd.org/cm_data/urinary_iodine-method_a.pdf. Accessed February 14th 2019.

[14] Seal Broshure. Available at: http://www.seal-us.com/Portals/0/AA3%20CSM/5%20-%20AA3%20Training%20-%20HR%20System.pdf. Accessed February 14th 2019.

[15] ClinChek. Control Urine Control lyophilised / Kontrollurin lyophilisiert ClinChek - Control. 2010. Available at: https://www.recipe.de/pdf/Datenblaetter%20ClinChek/884749_1227-1.pdf Accessed August 19th 2018.

[16] Centers for Disease Control and Prevention (CDC). Ensuring the Quality of Urinary Iodine Procedures (EQUIP). Available at: https://www.cdc.gov/labstandards/equip.html. Accessed February 14th 2019.

[17] Clinical and Laboratory Standards Institute (CLSI). User Verification of Precision and Estimation of Bias, 2nd edition. Document EP15-A2. Wayne: CLSI; 2005.

[18] The Royal College of Pathologists of Australasia Quality Assurance Programs (RCPAQAP). Allowable Limits of Performance. Available at: http://www.rcpaqap.com.au/docs/2014/chempath/ALP.pdf. Accessed August 6th 2018.

[19] Armbruster DA Alexander DB Sample to sample carryover : A source of analytical laboratory error and its relevance to integrated clinical chemistry / immunoassay systems. Clin Chim Acta. 2006;373:37–43. 10.1016/j.cca.2006.04.022 16777083

[20] Tesija Kuna A Dukic L Norac Gabaj N Miler M Vukasovic I Langer S Comparison of enzymatic assay for HbA1c measurement (Abbott Architect) with capillary electrophoresis (Sebia Minicap Flex piercing analyser). Lab Med. 2018;49: 231–8. 10.1093/labmed/lmx090 29528429

[21] Clinical and Laboratory Standards Institute (CLSI). Evaluation of the linearity of quantitative measurement procedures: a statistical approach, 2nd ed. Document EP06. Wayne: CLSI; 2003.

[22] Clinical and Laboratory Standards Institute (CLSI). EP09-A3 Measurement Procedure Comparison and Bias Estimation Using Patient Samples, 3rd ed. Document EP09-A3. Wayne: CLSI; 2014.

[23] Bilić-Zulle L Comparison of methods: Passing and Bablok regression. Biochem Med (Zagreb). 2011;21:49–52. 10.11613/BM.2011.010 22141206

[24] Gnat D Dunn DA Chaker S Delange F Vertongen F Dunn TJ Fast Colorimetric Method for Measuring Urinary Iodine. Clin Chem. 2003;49:186–8. 10.1373/49.1.186 12507981

[25] May W Wu D Eastman C Bourdoux P Maberly G Evaluation of automated urinary iodine methods: problems of interfering substances identified. Clin Chem. 1990;36:865–9. 2357823

[26] Seal AJ Creeke P Gnat D Abdalla F Mirghani Z Excess dietary iodine intake in long-term African refugees. Public Health Nutr. 2006;9:35–9. 10.1079/PHN2005830 16480531

[27] Bier D Rendl J Ziemann M Freystadt D Reiners C Methodological and analytical aspects of simple methods for measuring iodine in urine. Comparison with HPLC and Technicon Autoanalyzer II. Exp Clin Endocrinol Diabetes. 1998;106:S27–31. 10.1055/s-0029-1212042 9865550

[28] Ohashi T Yamaki M Pandav CS Karmarkar MG Irie M Simple microplate method for determination of urinary iodine. Clin Chem. 2000;46:529–36. 10759477

[29] Jooste PL Strydom E Methods for determination of iodine in urine and salt. Best Pract Res Clin Endocrinol Metab. 2010;24:77–88. 10.1016/j.beem.2009.08.006 20172472

