# Corrigendum to: Analytical evaluation of the new Seal Autoanalyzer 3 High Resolution for urinary iodine determination

**DOI:** 10.11613/BM.2019.031201

**Published:** 2019-10-15

**Authors:** Valentina Vidranski, Maja Franceschi, Dražena Krilić, Tomislav Jukić, Ivan Mihaljević, Zvonko Kusić

**Affiliations:** 1 Department for Oncology and Nuclear Medicine, Sestre milosrdnice University Hospital Center, Zagreb, Croatia; 2 Faculty of Medicine Osijek, Josip Juraj Strossmayer University of Osijek, Osijek, Croatia; 3 Department of Nuclear Medicine, School of Medicine, University of Zagreb, Zagreb, Croatia; 4 Department for Nuclear Medicine and Radiation Protection, University Hospital Center Osijek, Osijek, Croatia; 5 Croatian Academy of Sciences and Arts, Zagreb, Croatia

This is a correction of of Biochem Med (Zagreb)2019;29(2):020711. DOI: https://doi.org/10.11613/BM.2019.020711

Since the publication of the article, the authors have noticed that one of the authors in the byline was missing an affiliation. The correct byline is presented above. The authors apologize for any inconvenience caused to the readers.

